# The Association between Birthweight and Use of Cardiovascular Medications: The Role of Health Behaviors

**DOI:** 10.3390/jcdd10100426

**Published:** 2023-10-15

**Authors:** Minjia Mo, Robert Thiesmeier, George Kiwango, Christian Rausch, Jette Möller, Yajun Liang

**Affiliations:** 1 Department of Neurobiology, Care Sciences and Society, Karolinska Institutet, 14183 Stockholm, Sweden; 2 Department of Global Public Health, Karolinska Institutet, 17177 Stockholm, Sweden; 3 Department of Physiology, Muhimbili University of Health and Allied Sciences, 17105 Dar es Salaam, Tanzania

**Keywords:** birthweight, cardiovascular medications, fetal growth, health behaviors, life course

## Abstract

Background: There is limited evidence on the effect of low birthweight on the use of cardiovascular medications and the role of health behaviors. This study aims to determine the independent effect of low birthweight and its combination with adult health behaviors on the number of dispensed cardiovascular medications. Methods: We included 15618 participants with information on birthweight and self-reported health behaviors. Dispensed cardiovascular medications were identified from the Prescribed Drug Register based on a three-digit level Anatomical Therapeutic Chemical classification code (C01 to C10 and B01) and categorized into 0, 1, and ≥2 different types of medications. We applied multinomial logistic regression models estimating odds ratios (ORs) and 95% confidence intervals (CIs). Results: Participants with low birthweight had a higher estimated OR of using ≥2 types of cardiovascular medications (OR = 1.46, 95% CI = 1.06, 2.01). Further, an increased risk for using ≥2 types of cardiovascular medications was found in participants with poor health behaviors for normal (OR = 2.17, 95% CI = 1.80, 2.62) and high (OR = 1.84, 95% CI = 1.29, 2.62) birthweight. The strongest effect on using ≥2 types of cardiovascular medications was found for low birthweight and poor health behaviors (OR = 3.14, 95% CI = 1.80, 5.50). Conclusion: This cohort study provides evidence that low birthweight increases the risk of using more types of cardiovascular medications in adulthood. This study also suggests that ideal health behaviors reduce this risk.

## 1. Background

Cardiovascular medications are among the most frequently prescribed medications in Sweden [1]. However, despite their use for the prevention and treatment of cardiovascular diseases (CVDs), the increasingly common prescription and use of cardiovascular medications is thought to cause multifaceted problems such as increases in polypharmacy [2,3], harmful drug–drug interactions, and adverse drug events [4]. Certain cardiovascular medications have been linked to ill-health outcomes such as cancer [5,6,7,8,9], neuropsychiatric symptoms [10], and depression [11], although there is no consensus on the direction of evidence in the medical community. In addition, it has been indicated that antihypertensives (e.g., beta-blockers, ACE-I), diuretics, and antiarrhythmics (e.g., digoxin) are amongst the most frequent medications thought to cause medication harm leading to increased hospitalizations, prolonged hospital stays, and adverse health outcomes for patients [12].

Besides traditional risk factors occurring during adolescence and adulthood, there are strong indications that fetal and infant life could be critical periods for the development of CVDs and subsequent cardiovascular medication use in mid-to-late adulthood [13]. Low birthweight (LBW; <2.5 kg) has been shown to be associated with congenital heart diseases [14,15] and cardiovascular morbidity and mortality, based on a diverse body of evidence, including various study designs dating back to 1989 [16,17,18,19,20,21,22].

Despite a wealth of evidence on LBW and CVDs, few studies have assessed the effect of LBW on the use of cardiovascular medications. Most of the previous studies have focused on the onset risk of CVDs, and very few on the use of cardiovascular medications, which can be a primary and secondary prevention of CVDs. Due to the high burden and adverse consequences of using cardiovascular medications, it is relevant to provide evidence from a life-course perspective on the management of cardiovascular medications through identifying the risk factors in early life. Studying the association between fetal growth and the use of cardiovascular medications has relevant public health implications for the primary and secondary prevention of CVDs. In addition to birthweight constituting a popular public health intervention target, numerous studies suggest that adopting a healthy lifestyle can attenuate the risk of CVDs due to LBW [22,23,24]. Yet, due to the lack of evidence on the association between fetal growth and the use of cardiovascular medications, the interplay between LBW, health behaviors, and cardiovascular medication use is not well understood.

Based on previous studies, we hypothesize that LBW may increase the risk of using a higher number of dispensed cardiovascular medications and that a healthy lifestyle can potentially mitigate the risks that may be present due to LBW. Therefore, the aim of this study is to determine the individual effect of LBW and health behaviors and their combinations on the risk of using a higher number of cardiovascular medications later in life.

## 2. Methods

### 2.1. Study Design

The study population was based on the Stockholm Public Health Cohort (SPHC) in Sweden, which has been described in details elsewhere [25] and is described briefly herein. The SPHC was established within the framework of the Stockholm County Council public health surveys. The baseline survey was conducted in 2002 with three consecutive follow-ups in 2007, 2010, and 2014. New participants entered the study in 2006 and 2010. In total, three sub-cohorts were identified for this study: sub-cohort 2002 (*n* = 31,182), sub-cohort 2006 (*n* = 34,707), and sub-cohort 2010 (*n* = 30,767). The baseline surveys of three cohorts were conducted in 2002, 2006, and 2010, and the corresponding response rates were 62%, 61%, and 56%, respectively [25]. Because the Medical Birth Register in Sweden was established in 1973, those born in 1973 and afterward with available information from the Medical Birth Register were thus included in the analysis. Therefore, the final analytical sample in this study included 15,618 participants: 2889 for sub-cohort 2002, 6203 for sub-cohort 2006, and 6526 for sub-cohort 2010. The participants were followed until the end of 2018.

### 2.2. Data Collection

Data were collected through self-reported questionnaires (postal or web-based), complementary telephone interviews, and further linkage to several national health and administrative registries including the Medical Birth Register (including birthweight, gestational age, and maternal CVDs), Swedish Prescribed Drug Register (2005 and onwards) (including dispensation date and medications), and Integrated Database for Labor Market Research (including sociodemographic factors).

### 2.3. Birthweight

Information on birthweight was extracted from the Medical Birth Register, which started in 1973. Birthweight was categorized into three mutually exclusive groups: <2.5 kg, 2.5–4.0 kg, and >4.0 kg, indicating LBW, normal birthweight (NBW), and high birthweight (HBW), respectively [19,21,26].

### 2.4. Health Behaviors

Data on health behaviors were self-reported in the questionnaires, which were worded the same for each cohort. Four behavioral factors, namely smoking, physical activity, dietary factors, and body mass index, were included to define behavioral health metrics [27]. For each of the four components, participants were categorized into one of the following three levels: poor (score = 0), intermediate (score = 1), and ideal (score = 2) [28]. A poor level of smoking was defined as current smoking, an intermediate level of smoking was defined as former smoking or having quit smoking less than one year ago, and an ideal level of smoking was defined as never smoking or having quit more than one year ago. A poor level of physical activity was defined as no regular physical activity, an intermediate level of physical activity was defined as walking or cycling less than 20 min per day or engaging in physical exercise equivalent to no more than two hours per week, and an ideal level of physical activity was defined as walking or cycling more than 20 min per day or exercising more than two hours per week. A poor level of diet was defined as eating fruit less than once per week, an intermediate level of diet was defined as eating fruit a few times per week, and an ideal level of diet was defined as eating fruit every day. A poor level of body mass index was defined as having a body mass index ≥30 kg/m^2^, an intermediate level of body mass index was defined as a body mass index being 25–29.9 kg/m^2^, and an ideal level of body mass index was defined as a body mass index <25 kg/m^2^. In the case of missing behaviors at baseline, data from the subsequent follow-up were used as an indication. The overall score of behavioral health metrics was calculated as the sum of all scores of the four components and categorized into poor (total score ≤ 4), intermediate (5–6), and ideal (≥ 7).

### 2.5. Dispensed Cardiovascular Medications

Information on dispensed cardiovascular medications was extracted from the Prescribed Drug Register from July 2005 to December 2018. The type of specific cardiovascular medications was identified based on the three-digit level Anatomical Therapeutic Chemical (ATC) Classification System codes and included all medications ranging from C01 to C10 (including medications for cardiac therapy, antihypertensives, diuretics, peripheral vasodilators, vasoprotectives, beta-blocking agents, calcium channel blockers, agents acting on the renin–angiotensin system, and lipid-modifying agents). In addition, we included B01 medications (antithrombotic agents). Cardiovascular medications with the same ATC code were counted as one despite them being dispensed several times during the study period. The types of specific cardiovascular medications were identified and categorized into three groups: 0, 1, and ≥2.

### 2.6. Covariates

Sociodemographic factors (e.g., sex, age, education, marital status) were identified from the register. Formal education was categorized into the following three groups: compulsory school (≤ 9 years), upper secondary school (9–11 years), and university and above (≥12 years). Marital status was categorized into married and unmarried groups. From the SPHC questionnaire, alcohol drinking was assessed using the following question: “During the last 12 months, how often have you, on the same occasion, consumed alcoholic beverages equivalent to at least: 1 bottle of wine or 5 glasses of spirits or 4 cans of strong beer or 6 cans of medium-strength beer.” Alcohol drinking was grouped into “never”, “1–6 times/year”, and “at least once per month”. Gestational age and maternal CVD were retrieved from the Medical Birth Register. Maternal CVDs were defined based on the International Classification of Diseases (ICD) codes: ICD-9 codes 390-459 or ICD-10 codes 100-199.

### 2.7. Statistical Analysis

Baseline characteristics were calculated as mean and standard deviation (SD) for continuous variables and as number and percentage (%) for categorical variables. Multinomial logistic regression analysis was used to estimate the odds ratios (ORs) and 95% confidence intervals (CIs). First, the association between birthweight and the number of dispensed medications was examined. Second, the effect of the combination of birthweight and health behaviors on cardiovascular medications was assessed. A joint variable between birthweight (low, normal, high) and health behaviors (poor, intermediate, ideal) was generated and added as the independent variable in the multinomial logistic regression. Two models were presented: a crude and an adjusted model. In the adjusted model, we adjusted for sex, education, gestational age, maternal CVDs, and if applicable, baseline age, and alcohol drinking. The statistical analysis was performed using Stata 17 software.

## 3. Results

### 3.1. Participant Characteristics

The baseline characteristics of the participants according to the three birthweight categories (i.e., low, normal, high) are presented in Table 1. Overall, 3.9% and 15.3% had LBW and HBW, respectively. Approximately 57.5% of the study participants were women, and the proportion of women was lowest among those with HBW (*p* < 0.001). Participants with LBW had the lowest gestational age; the lowest prevalence of normal body mass index (25–29.9 kg/m^2^); and the highest prevalence of maternal CVDs, never drinking alcohol, and a poor level of smoking (all *p* < 0.01). There was no statistically significant difference in age at participation, education, diet, physical activity, and behavioral health metrics between these groups (all *p* > 0.10). During the follow-up period, the cumulative incidence of CVDs was 4.7% among total participants, and the cumulative incidence of CVDs was 6.5%, 4.7%, and 4.0% for participants with LBW, NBW, and HBW, respectively. The cumulative incidence of CVDs was highest among those with LBW (*p* = 0.03).

### 3.2. Association between Birthweight and Use of Cardiovascular Medications

Table 2 displays the association between birthweight and prescribed cardiovascular medications. A total of 11,225, 3261, and 1132 participants used 0, 1, and ≥2 types of different cardiovascular medications, respectively. Compared to participants with NBW, those with LBW had a significantly higher risk of using ≥2 types of cardiovascular medications (crude model: OR = 1.36; 95% CI = 1.03, 1.81), and the association remained statistically significant after the adjustment for gestational age, maternal CVDs, education, and drinking (adjusted model: OR = 1.46; 95% CI = 1.06, 2.01). In the crude model, HBW was found to have a protective effect on using 1 type (OR = 0.82; 95% CI = 0.74, 0.92) and using ≥2 types of cardiovascular medications (OR = 0.77; 95% CI = 0.64, 0.92), but the associations were not statistically significant after the adjustment of covariates.

As shown in Table 3, the three most common types of medications were antithrombotic agents (B01, 7.71%), vasoprotectives (C05, 7.68%), and beta-blocking agents (C07, 5.95%).

In a stratified analysis by CVDs, we found that among participants diagnosed with CVDs, compared to those with NBW, LBW was associated with a higher risk of using more than two types of CVD medications (OR = 3.80; 95% CI = 1.49, 9.70) after controlling for confounders (Table 4). However, among individuals without CVDs, the association between LBW and using cardiovascular medications was not statistically significant, although there was a trend of positive association between LBW and using two or more types of cardiovascular medications (OR = 1.11; 95% CI = 0.75, 1.62). No significant association was found for participants with HBW compared to those with NBW.

### 3.3. Combination of Birthweight and Behaviors

**Figure 1:**
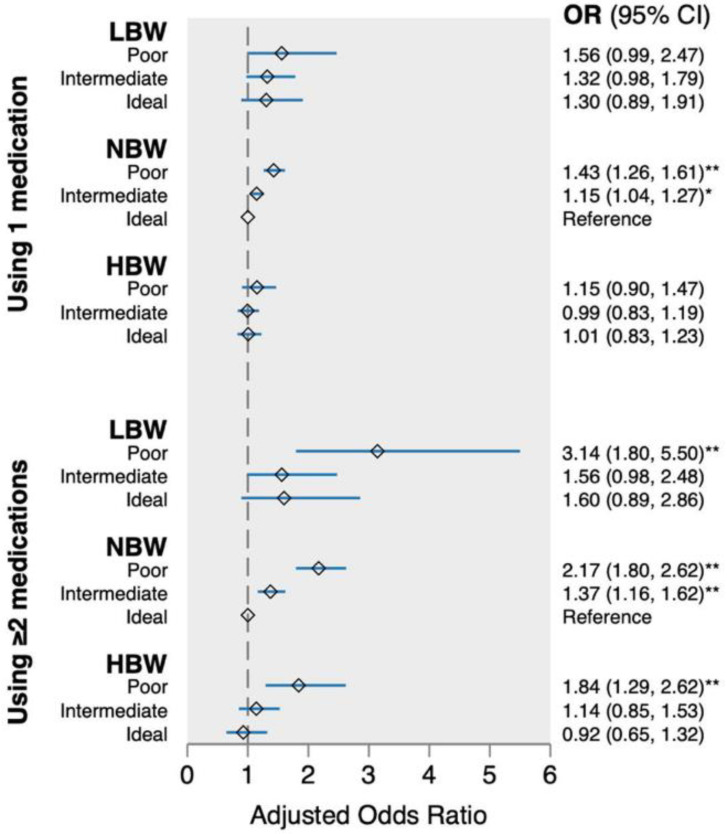
Combination effect of birthweight and health behaviors on dispensed cardiovascular medications (*n* = 15,618). The odds ratio (95% CI) was adjusted for sex, baseline age, maternal CVD, gestational age, education, and alcohol drinking. *Abbreviations: LWB, low birthweight; NBW, normal birthweight; HBW, high birthweight; OR, odds ratio; CI, confidence interval.* * *p*-value < 0.05; ** *p*-value < 0.001.

Figure 1 depicts the adjusted combination effect between birthweight and health behaviors on dispensed cardiovascular medications. Compared with people with NBW and ideal health behaviors, those with NBW and poor/intermediate health behaviors had higher odds of using one type of cardiovascular medication, and the OR (95% CI) was 1.43 (1.26, 1.61) and 1.15 (1.04, 1.27) for NBW–poor and NBW–intermediate behaviors, respectively.

Compared with people with NBW and ideal health behaviors, the odds of using ≥2 types of cardiovascular medications were statistically significant for all groups with poor health behaviors regardless of birthweight. The OR (95% CI) of using ≥2 types of cardiovascular medications was 3.14 (1.80, 5.50) among those with LBW and poor health behaviors, 2.17 (1.80, 2.62) among those with NBW and poor behaviors, and 1.84 (1.29, 2.62) among those with HBW and poor health behaviors. Additionally, there was a 37% increased risk of using ≥2 types of cardiovascular medications among those with NBW and intermediate health behaviors (OR = 1.37; 95% CI = 1.16, 1.62). The direction and magnitude of the effect were similar for men and women.

## 4. Discussion

In this cohort study, we found that LBW was associated with a higher risk of using ≥2 types of cardiovascular medications in adulthood, and the association was more evident among those with CVDs. Compared with participants with NBW and ideal behaviors, participants with LBW and poor behaviors had a three-fold increase in the odds of using ≥2 types of cardiovascular medications. Further, we found that poor health behaviors play a leading role in the risk of using multiple types of cardiovascular medications independent of birthweight.

Despite a lack of evidence from previous studies, our findings, however, seem to be in accordance with studies of a similar design and related outcome, such as CVDs. We found that those born with LBW had a higher risk of using ≥2 types of cardiovascular medications in adulthood. The potential underlying mechanisms might be in line with those highlighted in previous research showing that fetal growth restrictions, in particular LBW, have long-term physiological and developmental effects that predispose an individual to, and increase the risk of, diseases in later life. Thus, fetal growth restriction may increase the risk of adulthood CVDs for which cardiovascular medications are prescribed. Our findings showed that the risk of using more types of cardiovascular medications in association with LBW was more evident among those participants with CVDs. This finding further confirmed the role of CVDs in the pathway between LBW and the use of cardiovascular medications. While assessing cardiovascular medication use, it can be assumed that a large burden of CVDs in the population is captured. In addition, the use of multiple types of cardiovascular medication can be considered as a surrogate for the severity or complexity of CVDs. However, cardiovascular medications might also be used to treat diseases other than cardiovascular conditions, although the proportion of people using cardiovascular drugs for non-cardiovascular indications is low [29,30,31].

Previous studies have found that fetal growth restrictions may interact with later lifestyles to increase adulthood risk for CVDs [24]. Li et al. [32] reported an interaction between unfavorable lifestyles and LBW in cardiometabolic diseases in a Swedish twin study. Similarly, Wang et al. [22] showed an additive interactive effect between LBW and poor lifestyles in three large North American cohort studies. We found that favorable health behaviors can mitigate the initial risk of LBW, which is in line with previous findings from Wang et al. [22] suggesting that adapting to a healthier lifestyle is pivotal in mitigating the risk of later-life diseases originating due to fetal growth restrictions. Moreover, the findings of the current study demonstrate an independent effect of health behaviors, regardless of initial risk due to LBW, in adults in relation to the use of cardiovascular medications. Our results strongly indicate that adapting ideal health behaviors in young adulthood can mitigate the risk of using multiple cardiovascular medications regardless of the initial risk due to LBW.

## 5. Strengths and Limitations

This study benefits from a longitudinal cohort study design, a long follow-up, minimal loss to follow-up due to the linkage to register data on prescribed medications, a consistent study protocol for different waves, and reliable information on birth markers and medication based on registers.

Despite its strengths, this study is subject to several limitations. First, the Prescribed Drug Register was started in June 2005. Thus, this study was unable to identify the prescriptions of cardiovascular medications before 2005. Hence, this might lead to an underestimation of the risk of using cardiovascular medications. Second, we did not consider the dose and frequency of dispensed medications due to the lack of data; thus, we are unable to identify the exact and detailed information on the use of cardiovascular medications. Third, we were unable to consider other comorbidities except for CVDs in the analysis. Since we do not have information on the indications for the dispensed medications, we have to acknowledge that this increases the risk of outcome misclassification as the medications could be used for other comorbidities. Fourth, this study only included health behaviors at baseline, and if missed, those measured in the wave following baseline were used. These health behaviors were one-time measurements, and thus, changes in health behaviors were not considered. Future studies with more detailed analyses of behavioral changes are needed to give a more detailed insight into the role of health behaviors. Fifth, the behavioral factors used in this study were self-reported, and there was a lack of details about specific behaviors such as dietary patterns. The self-reported health behaviors might lead to an underestimation of poor health behaviors resulting in an underestimation of the association between poor health behaviors and the use of cardiovascular medications. Sixth, we only included education as an indicator of social class, which might not be a sufficient measure. Last, this study is subject to low statistical power in some sub-group analyses assessing the combination effect between birthweight and health behaviors. However, it is argued that the observed pattern of the combination effect between birthweight and health behaviors on the use of cardiovascular medication is of a higher clinical and public health relevance than a sufficiently large statistical power to detect an effect [33].

## 6. Conclusions

This study found evidence that the joint impact of LBW and poor health behaviors increases the risk of using multiple dispensed cardiovascular medications in adulthood. The results highlight the pivotal role of health behaviors in health promotion and prevention to reduce the burden of using cardiovascular medications in adults.

## Figures and Tables

**Table 1:** Characteristics of study participants of the Stockholm Public Health Cohort.

	Total (*n* = 15,618)	LBW (*n* = 605)	NBW (*n* = 12,627)	HBW (*n* = 2386)	*p*-Value
Cohort					0.15
Sub-Cohort 2002	2889 (18.5)	108 (17.9)	2358 (18.7)	423 (17.7)	
Sub-Cohort 2006	6203 (39.7)	240 (39.7)	5053 (40.0)	910 (38.1)	
Sub-Cohort 2010	6526 (41.8)	257 (42.5)	5216 (41.3)	1053 (44.1)	
Age at participation	26.5 (5.4)	26.2 (5.5)	26.5 (5.4)	26.7 (5.4)	0.17
Female	8987 (57.5)	364 (60.2)	7571 (60.0)	1052 (44.1)	<0.001
Gestational age	39.6 (1.8)	35.6 (3.2)	39.6 (1.6)	40.5 (1.3)	<0.001
Maternal CVDs	197 (1.3)	22 (3.6)	153 (1.2)	22 (0.9)	<0.001
Educational level					0.15
Compulsory school	3065 (19.6)	134 (22.1)	2494 (19.8)	437 (18.3)	
Upper secondary school	7130 (45.7)	286 (47.3)	5759 (45.6)	1085 (45.5)	
University and above	5372 (34.4)	184 (30.4)	4332 (34.3)	856 (35.9)	
Missing	51 (0.3)	1 (0.2)	42 (0.3)	8 (0.3)	
Body mass index					<0.001
≥30 kg/m^2^	890 (5.7)	34 (5.6)	689 (5.5)	167 (7.0)	
25–29.9 kg/m^2^	3092 (19.8)	116 (19.2)	2431 (19.3)	545 (22.8)	
<25 kg/m^2^	11,636 (74.5)	455 (75.2)	9507 (75.3)	1674 (70.2)	
Alcohol drinking					<0.001
At least once a month	10,601 (67.9)	381 (63.0)	8483 (67.2)	1737 (72.8)	
1–6 times per year	3730 (23.9)	168 (27.8)	3105 (24.6)	457 (19.2)	
Never	1287 (8.2)	56 (9.3)	1039 (8.2)	192 (8.0)	
Diet					0.54
Poor	1741 (11.1)	63 (10.4)	1397 (11.1)	281 (11.8)	
Intermediate	7798 (49.9)	318 (52.6)	6288 (49.8)	1192 (50.0)	
Ideal	6079 (38.9)	224 (37.0)	4942 (39.1)	913 (38.3)	
Smoking					0.008
Poor	1792 (11.5)	86 (14.2)	1477 (11.7)	229 (9.6)	
Intermediate	1154 (7.4)	41 (6.8)	939 (7.4)	174 (7.3)	
Ideal	12,672 (81.1)	478 (79.0)	10,211 (80.9)	1983 (83.1)	
Physical activity					0.15
Poor	4274 (27.4)	184 (30.4)	3430 (27.2)	660 (27.7)	
Intermediate	6202 (39.7)	230 (38.0)	5064 (40.1)	908 (38.1)	
Ideal	5142 (32.9)	191 (31.6)	4133 (32.7)	818 (34.3)	
Behavioral health metrics					0.13
Poor (≤4)	3086 (19.8)	117 (19.3)	2473 (19.6)	496 (20.8)	
Intermediate (5–6)	7198 (46.1)	304 (50.2)	5803 (46.0)	1091 (45.7)	
Ideal (≥7)	5334 (34.2)	184 (30.4)	4351 (34.5)	799 (33.5)	
Cumulative incidence of CVDs	729 (4.7)	39 (6.5)	595 (4.7)	95 (4.0)	0.03

Abbreviations: LBW, low birthweight; NBW, normal birthweight; HBW, high birthweight; CVDs, cardiovascular diseases. Values are presented as mean (SD) for continuous variables and as number (%) for categorical variables.

**Table 2:** The association between birthweight and dispensed cardiovascular medications.

Birthweight	No. of Cases	Odds Ratio (95% CI) for Using 1 Type of Medication		No. of Cases	Odds Ratio (95% CI) for Using ≥2 Types of Medications	
		Crude	Adjusted *		Crude	Adjusted *
Low	136	1.11 (0.91, 1.36)	1.22 (0.98, 1.53)	58	1.36 (1.03, 1.81)	1.46 (1.06, 2.01)
Normal	2683	Ref	Ref	931	Ref	Ref
High	442	0.82 (0.74, 0.92)	0.89 (0.79, 1.00)	143	0.77 (0.64, 0.92)	0.85 (0.71, 1.03)

Abbreviations: OR, odds ratio; CI, confidence interval. Participants not being prescribed any cardiovascular medications were considered as a reference group. * Adjustments were made for maternal cardiovascular disease, gestational age, sex, and education.

**Table 3:** The frequencies of cardiovascular medications.

ATC Code	CVD Medications	*n*	Percent, %
B01	Antithrombotic agents	1204	7.71
C05	Vasoprotectives	1200	7.68
C07	Beta-blocking agents	930	5.95
C01	Cardiac therapy	408	2.61
C09	Agents acting on the renin–angiotensin system	243	1.56
C03	Diuretics	153	0.98
C10	Lipid-modifying agents	113	0.72
C08	Calcium channel blockers	104	0.67
C02	Antihypertensives	38	0.24

**Table 4:** The association between birthweight and dispensed cardiovascular medications after stratification by cardiovascular diseases (*n* = 15,618).

Birthweight	No. of Cases	Odds Ratio (95% CI) for Using 1 Type of Medication		No. of Cases	Odds Ratio (95% CI) for Using ≥2 Types of Medications	
		Crude	Adjusted *		Crude	Adjusted *
CVD patients						
Low	8	0.90 (0.36, 2.29)	1.02 (0.34, 3.12)	20	2.36 (1.10, 5.05)	3.80 (1.49, 9.70)
Normal	186	Ref	Ref	178	Ref	Ref
High	26	0.70 (0.42, 1.18)	0.75 (0.44, 1.28)	23	0.65 (0.38, 1.11)	0.65 (0.38, 1.14)
Non-CVD participants						
Low	128	1.13 (0.92, 1.38)	1.24 (0.99, 1.56)	38	1.11 (0.79, 1.56)	1.11 (0.75, 1.62)
Normal	2497	Ref	Ref	753	Ref	Ref
High	416	0.83 (0.74, 0.94)	0.90 (0.80, 1.02)	120	0.80 (0.65, 0.97)	0.91 (0.74, 1.11)

Abbreviations: CVD, cardiovascular disease; OR, odds ratio; CI, confidence interval. Participants not being prescribed any cardiovascular medications were considered as a reference group. * Adjustments were made for maternal cardiovascular disease, gestational age, sex, and education.

## Data Availability

The datasets used during the current study are available from the corresponding author upon reasonable request.

## Abbreviations

CVDs: Cardiovascular Diseases; LBW: Low Birthweight; SPHC: Stockholm Public Health Cohort; NBW: Normal Birthweight; HBW: High Birthweight; ATC: Anatomical Therapeutic Chemical; ICD: International Classification of Diseases; SD: Standard Deviation; OR: Odds Ratio; CI: Confidence Interval.
